# Up-regulation of IGF-1 in the frontal cortex of piglets exposed to an environmentally enriched arena

**DOI:** 10.1016/j.physbeh.2017.02.030

**Published:** 2017-05-01

**Authors:** Sarah M. Brown, Rebecca Peters, Alistair B. Lawrence

**Affiliations:** a University of Edinburgh, Roslin Institute, Penicuik EH25 9RG, United Kingdom; b SRUC, West Mains Road, Edinburgh EH9 3JG, United Kingdom

**Keywords:** Enrichment, Behaviour, Gene expression, Frontal cortex, IGF-1

## Abstract

Environmental enrichment (EE) is widely used in the life sciences to study effects of environment on the brain. In pigs, despite lack of EE being a key welfare issue there is little understanding of brain effects of EE in pigs. This project aimed to study the effects of exposure to an EE arena on piglet behaviours and on brain gene expression levels with a focus on *IGF-1* and related genes. Eight litters of large white × landrace × Hampshire piglets were farrowed and raised in a free farrowing system (PigSAFE). At 42 days of age, 6 piglets per litter were given access to an enriched arena with plentiful peat, straw and space, (in groups of 4 made up of stable pairs) for 15 min per day on 5 consecutive days to allow them to habituate to the apparatus. Piglet behaviours were recorded in the arena for 15 min periods on 3 consecutive days. On the final day only one pair of test piglets per litter was given access to the arena. Brain tissue was collected within 45 min of the test from piglets exposed to the arena on the day and their non-exposed littermate controls. RNA was extracted from the frontal cortex and QRT-PCR for selected genes run on a Stratgene MX3005P. In both the home pen and the EE arena litters spent the largest proportion of time engaging in foraging behaviour which was significantly increased in the enriched arena (*t*_7_ = 5.35, df = 6, *p* = 0.001). There were decreases in non-running play (*t*_7_ = 4.82, *p* = 0.002) and inactivity (*t*_7_ = 4.6, *p* = 0.002) in the arena. A significant fold change increase (FC = 1.07, *t* = 4.42, *p* = 0.002) was observed in *IGF-1* gene expression in the frontal cortex of piglets exposed to the enriched arena compared to those not exposed on the day of culling. No change in expression was observed in *CSF1*, the *IGF-1* receptor gene nor in any of the binding proteins tested (*IGFBP1-6*). There was a weak tendency for increased expression of the neurotrophic factor *BDNF1* (fold change: 1.03; *t*_7_ = 1.54, *p* = 0.1). We believe this work is the first to explore effects of EE on pig brain physiology and development, and also points to a potential role for *IGF-1* in brain effects of EE.

## Highlights

• *IGF-1* gene expression upregulated in frontal cortex of piglets after EE protocol.
• Trend towards similar increase in BDNF post enrichment.
• Evidence of potential CNS benefits of EE.
• No difference in exploration or walking behaviour in enriched arena vs home pen.
• Species typical behaviours (rooting/foraging) increased in enriched arena while inactivity decreased.

## 1 Introduction

Environmental enrichment (EE) refers to providing increased environmental complexity to housed (*e.g.* farmed, laboratory) animals, and has been defined as an environmental manipulation that improves the biological functioning of the animal [1]. In general EE involves keeping animals in larger cages or pens in groups with nesting materials, objects and in the case of rodents with running wheels (*e.g.* [2], [3]). Thus EE generally provides for a greater range of behaviours including social interactions, species typical behaviour such as digging and nest-building and exercise.

It is many years since EE was first demonstrated to have what are regarded as positive effects on brain and behaviour development (*e.g.* [4], [5]). Since that time studies have continued to use EE as a means of studying the effects of environment on brain morphology and function, for example studying the role of EE in stimulating neurogenesis and cognitive function in relation to the hippocampus [6]. There has also been increasing interest in the role of EE in protecting against challenges such as anxiety [7] and ageing [8]. The components of EE which affect brain and behaviour remain under debate. There has been particular interest in the role of exercise as an aspect of EE. For example, in a study where exercise was dissociated from other aspects of EE, exercise was concluded to be the critical factor in mediating increased hippocampal neurogenesis [9]. Another study using an ‘alternating EE paradigm’ confirmed the importance of physical exercise to neurogenesis while suggesting that other components of EE had other effects including buffering against stress [10].

In terms of molecular mechanisms growth and neurotrophic factors are seen to play important roles in mediating the effects of EE. The exercise components of EE seem likely to be responsible for increased expression of vascular endothelial growth factor (VEGF) [11] and EE-induced VEGF has been associated with hippocampal neurogenesis and improved cognitive function [12]. Similarly EE has been shown to increase expression of neurotrophins; EE increased expression of both brain-derived growth factor (BDNF) and nerve growth factor (NGF) across several brain regions [13]. BDNF is thought to play an intrinsic role in terms of the improvements to synaptic plasticity and cognition observed with EE [6]. Insulin-like growth factor 1 (IGF-1) has also been implicated as a potential mediator of brain and behavioural effects of EE (*e.g.* [8]). IGF-1 is produced both in the periphery [14] and centrally within the brain [15], and plays an important role in neuronal differentiation, development and survival (*e.g.* [16]). IGF-1 exerts its effects on neurotrophic responses mainly through the IGF-1 receptor (IGF1R) [17], and may contribute to brain plasticity through stimulation of BDNF [16]. The literature mainly suggests that similar to VEGF, IGF-1 brain effects are stimulated via the exercise implicit in many EE regimes (*e.g.* [18]). However a study of the molecular basis of play behaviour (seen as a component of EE [3]), found *IGF-1* gene expression elevated in the frontal cortex of rats in response to play but not in response to a social defeat [19] suggesting that physical exercise *per se* may not be sufficient to induce brain IGF-1 expression.

In addition to its science relevance, EE is also a key concept in animal welfare given concern that housing for farm, laboratory and zoo animals often constrains performance of ‘natural behaviour’ [20]. EE also links to the growing interest in ‘positive welfare’, and that we should be concerned with providing animals with positive emotional daily experiences (*e.g.* [21]).

In this paper we report on the effects of EE on brain and behaviour in pigs. Most knowledge of the brain effects of EE comes from studies of adult rodents. The pig is both considerably more human-like in terms of anatomy and development than mice or rats [22], and information on the effects of EE in this species would thus have comparative value. The study of EE in pigs also has considerable relevance to pig welfare as pigs are often housed in conditions with limited space and a lack of materials with which to interact [23]. EU legislation requires that pigs are given access to EE in the form of materials to allow ‘proper investigation and manipulation activities’ (EU Directive; 2001/93/EC); there is concern that this directive is not widely enforced. We have focused on the potential for EE to elevate gene expression of growth and neurotrophic factors (*IGF1*, *BDNF*) given the evidence of their roles in brain effects of EE (see above). As the IGF-1 pathway has also been studied in detail following exposure to play [19] we examined effects of EE on gene expression of the *IGF-1* receptor and binding proteins. We have also looked for an effect of EE on colony stimulating factor 1 (*CSF1*). We carried out the study on juvenile pigs (this being the most relevant age in terms of welfare concerns). It is believed that neonatal macrophages are an important source of extra-hepatic IGF-1 [24], that microglia (brain macrophages) are the major source of brain IGF-1 [25] and that CSF1 may play a central role in stimulating macrophage production of IGF-1 [24]. We also report on the behavioural responses during exposure to EE which are often unreported in EE studies in rodents. We finally chose to explore the effects of a short-term exposure to EE, as this has previously been shown to have strong effects on a number of functional circuits [26]. In applied terms providing pigs with EE on farms for short periods could help circumvent some of the practical difficulties farmers have in providing EE materials to pigs.

In summary our aims were to test in the pig whether short-term exposure to EE induced elevations in gene expression for *IGF-1*, *BDNF* and *CSF1* and in the case of *IGF-1* for its receptor and binding proteins. We also report on the behavioural response to the EE exposure. To our knowledge this is the first study to report on growth and neurotrophic factors as potential brain mechanisms for effects of EE in a non-rodent large animal species.

## 2 Materials and methods

### 2.1 Ethical review

All work was carried out in accordance with the U.K. Animals (Scientific procedures) act 1986 under EU Directive 2010/63/EU following ethical approval by SRUC (Scotland's Rural College) Animal Experiments committee. All routine animal management procedures were adhered to by trained staff and health issues treated as required. All piglets were returned to commercial stock at the end of the study.

### 2.2 Animals and general experimental procedures

Post weaning behavioural observations and behavioural tests were carried out on litters from eight commercial cross-bred dams (Large White × Landrace); the boar-line was American Hampshire. Litters were born within a 72 h time window. Litter size was not standardised and was dependent on biological variation (11–13 piglets per litter in this study). Cross fostering was kept to a minimum and only performed where piglet welfare was considered at risk.

**Fig. 1:**
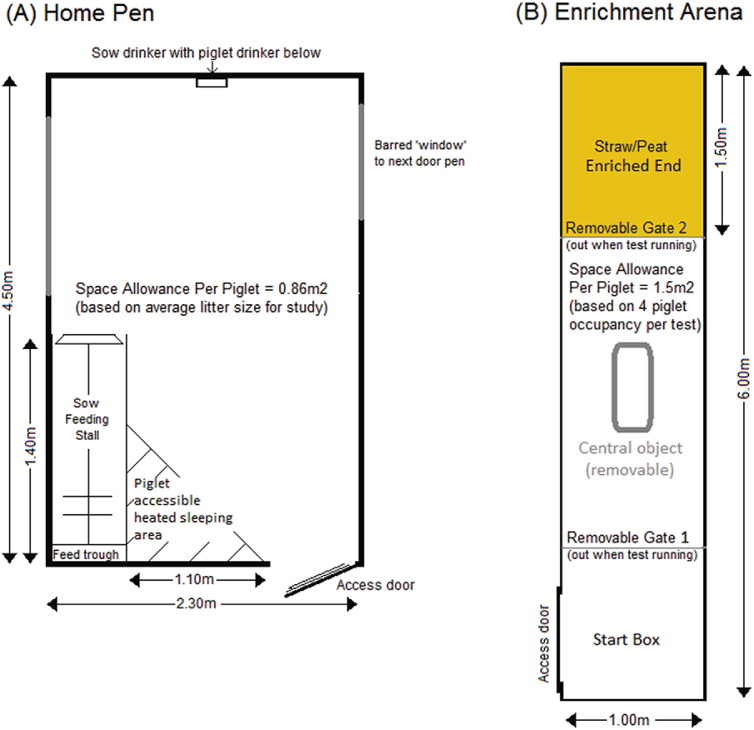
(a) Diagram of home pen for each litter; (b) Diagram of apparatus used in test events.

The experimental animals were housed in the *Pig and Sow Alternative Farrowing Environment* (PigSAFE) pens [27] from birth through to 8 weeks of age (4 weeks post weaning). PigSAFE (home) pens (Fig. 1a) allow species-specific behaviours in both the sow and the piglets, to be expressed by providing more space than conventional farrowing crates and the possibility for provision of bedding (straw; 1 kg per pen per day approximately provided at between 0830 and 0900). The PigSAFE pens used were partially slatted (approximately 2 m × 2.3 m slats at end opposite entry door) and straw was provided from entry door in solid floored area. Temperature within the unit was controlled in accordance to the Defra Code of Recommendations for the Welfare of Livestock [28]. Artificial lighting was maintained between the hours of 08:00–16:00 with low level night lighting ensuring Defra codes were adhered to. Piglet management included weighing at birth and a standard iron injection at day 3 post-partum. No teeth clipping or tail docking or castration was performed. Litter size was dependent on natural variation (range 10–12 piglets per litter in this group). Our previous work [29] has found no effect of litter size on the expression of the behaviours of interest in this system. At weaning sows were removed from the home pen and returned to the sow house while piglets were weighed, vaccinated against Porcine Circovirus and ear tagged for identification. Litters remained intact in the home pen until the end of the study (8 weeks of age) when remaining piglets were returned to commercial farm stock. Post wean diet was in the form of Primary Diets Prime Link Extra pelleted feed provided *ad libitum*.

### 2.3 Experimental design

Observations of behaviour in the home pen were performed on days 8, 11 and 13 post-weaning between the hours of 10:30 and 12:30. All piglets were marked on the back with an identity mark corresponding to their ear tag ID using a permanent marker between 08:00 and 09:30 on these days. Piglets were gently handled pre-weaning and so were used to the procedure. Behaviours were recorded using an ethogram based on previous work in play in pigs [29], with additional general behaviours of foraging, exploration, social non-play and walking added to record non-play behaviours (Table 1). Only those piglets from the litter that were subsequently observed in the arena were observed in the home pen.

Observations of behaviour in the enrichment (EE) arena (see Fig. 1b) were performed using the ethogram (Table 1) when the piglets were between 7 and 8 weeks of age (day 21–28 post weaning). The EE arena was solid floored throughout. Ten litres of peat and 1 kg of straw were provided at one end of the arena and replenished after every test. The arena was emptied of substrate and washed down daily. Six piglets per litter were introduced into the arena on a 10 min habituation schedule for three consecutive days. On the 4th day, piglets were marked as ‘stimulus’ or ‘test’ pigs (matched as closely as possible for weaning weight and sex) and run through the EE arena. All piglets were marked on the back with their own IDs between 08:00 and 09:30 daily. Other than moving to allow access to arena, test piglets were not handled differently to their littermates up to and including the point of sedation. All tests were carried out between 10:30 and 12:30. Test pigs were allocated to pair A or B at random and remained allocated to that pair for the duration of the study. Stimulus pigs were introduced and held at one end of the arena (enriched area) while test pigs were held at the opposite end (start box) for 30 s before release. Pigs had visual, auditory and olfactory access to their opposite pair throughout the test. Test pigs were then released and negotiated a short (2 m) runway past a central object before reuniting with the stimulus pigs at the opposite end. The central object was a plastic water container that occupied 0.06 m^2^ of floor space (0.22 × 0.22 m) and was removed once the piglets had passed it. Its purpose was to determine if piglets chose a specific side to pass the object, however as piglets were observed to pass the object on whichever side they were placed in the start box this analysis is not included. All four piglets were then left in the arena with barriers and central object removed for 15 min and behaviour recorded. At the end of the test, the pair of stimulus pigs were retained in the arena and the test pigs (pair A) replaced with the second pair of test pigs (pair B) from the same litter and the test repeated. Pair order for test pigs was swapped on each test. This was repeated on trial days 5 and 6, with only one test pair being run on day 6.

The EE arena was located in an empty corridor within the same room as the home pens (see Fig. 1b) and piglets were moved as needed using boards to minimise contact.

On day 6, after test pair A had completed the test one male piglet from the test pair B within the same litter was removed from the home pen (prior to the return of pair A to the pen) and sedated via intramuscular injection using a combination of medetomidine mydrochloride (Domitor 0.01 ml/kg), Ketamine (0.1 ml/kg), midazolam (Hypnovel 0.1 ml/kg) and azaperone (Stresnil 0.025 ml/kg) in a straw penned area in the same room but away from the other piglets. Piglets were left for 15 min to allow sedation to take full effect before being euthanized via intercardial injection with pentobarbital (Euthatal 0.7 ml/kg) for brain tissue collection. This method was devised by the consulting veterinary anaesthetist as the most effective and humane method of euthanasia for pigs of this age.

Within 15 min of completing the arena test, one male piglet from test pair A (which had experienced the test on day 6) was also removed from the home pen, sedated and euthanized using the same procedure. This provided brain tissue samples from 2 piglets per litter, both with experience of the enrichment arena, one within 24 h of brain collection and one within 45 min of brain collection. This timeline was used to give comparable results to those previously performed in rodents [26].

Piglet brains were removed whole and dissected over dry ice. Frontal cortex was snap frozen on dry ice and stored at − 80 °C prior to processing. Brain dissections were performed using the online pig brain atlas (http://www.anatomie-amsterdam.nl/sub_sites/pig_brain_atlas/start.htm) as a guide to gross structure with more precise dissections (as in Section 2.5) using information from [58].

### 2.4 Recording of behaviour

The animals were digitally recorded in their home pen using Sony LL20 low light cameras with infra-red and a Geovision GV-DVR (see above for schedule of recordings). Two cameras were set up per pen, one at the rear and one at the front to provide maximal coverage. Piglets were not visible when in the far corner of the heated sleeping area but could be seen at all other times. Behaviour was recorded using focal sampling with Noldus' *The Observer XT 11* (Noldus Information Technology bv, Wageningen, The Netherlands) software package. A coding scheme was created, relating each behaviour from the ethogram (Table 1) and every individual piglet with a specific key. Home pen play data was recorded as durations where appropriate. For short behaviours, such as hop or pivot, where it was not possible to get an accurate duration of one single movement, time was allocated as 1 s per count. Only piglets used in the arena tests were observed in the home pen. Behaviour of individual piglets in the enrichment arena was recorded from when the piglets had access to the whole arena (*i.e.* from the point that the test pigs mixed with the stimulus pigs) and stopped after 15 min. If the piglet moved out with the range of the recording equipment at any time in either the home pen or the arena it was recorded as being out of sight. The total period the piglet was out of sight was taken from the total observation period to give a visible time period of each piglet, which was then used as that piglet's observation time for calculating proportional behavioural time budgets. One observer completed all video analysis to remove any reliability issues relating to multiple observers.

### 2.5 Preparation of tissue samples for qPCR

Frontal cortex samples were removed from − 80 °C and placed in RNAlater (Ambion) which had been chilled to − 20 °C before being placed at 4 °C and allowed to thaw for 24 h under gentle agitation to assist in the perfusion of RNAlater through the tissue during thawing. The tissue was then further dissected to provide a small representative sample of superior frontal gyrus for RNA extraction.

RNA was extracted using the Qiagen RNeasy Midi Kit as per manufacturer's instructions. Samples were quantified using a Nanodrop 3300 (ThermoScientific). cDNA synthesis was carried out on 400 ng of RNA using the Affinity Script multi temperature cDNA synthesis kit (Agilent Technologies Part Number 200436) as per manufacturer's instructions. cDNA samples were also quantified using a Nanodrop 3300 (ThermoScientific) prior to PCR.

### 2.6 Quantitative real time PCR (qPCR)

qPCR was carried out on an MX3000P (Agilent Technologies) using Taqman FAM labelled assays and Agilent Brilliant III Ultra-Fast qPCR master mix as per manufacturer's instructions. HPRT1 (Applied Biosystems probe # Ss03388274_m1) and ACTB (Applied Biosystems probe # Ss03376081_u1) were used as reference genes. FAM tagged probes were supplied by Applied Biosystems as per Table 2. Cross species reactivity has been reported with some these probes (due to the highly conserved nature of the genes being studied) in the past. To prevent sample contamination all work areas and equipment were thoroughly cleaned with RNAzap and exposed to UV light before and after use. Researchers wore standard PPE and double gloved during sample collection and processing. No other tissue samples were processed in the same area of the laboratory for the duration of the gene expression work.

Samples were run in triplicate for both reference genes and genes of interest, and a calibrator sample (pooled) run on every plate to allow across plate comparison. Plates were initially held at 95 °C for 10 min before completing 40 cycles at 95 °C for 15 s and 60 °C for 1 min. The mean cycle threshold (Ct) value was calculated for each sample and a normalisation factor applied to correct for any errors in sample concentration by taking the geometric mean of the two control genes and dividing by the average geometric mean to create the normalisation factor for each sample, and then dividing the sample mean Ct by the sample normalisation factor. This normalised mean Ct was then rescaled to the plate calibrator (to allow across plate comparisons) by dividing the normalised mean Ct of the sample by the normalised mean Ct of the calibrator.

### 2.7 Statistical analysis

Basic statistics, normalisation and rescaling of qPCR values was carried out in Microsoft Excel. Further statistical analysis of qPCR (fold change: paired *t*-tests) and behavioural data (proportional changes: paired *t*-tests) was performed in Minitab 17 under licence to the University of Edinburgh. Bonferroni corrections for multiple testing (including behavioural and gene expression comparisons) provided a *p* value cut-off of 0.003. Home pen behaviour was only analysed for pigs used in the arena test, not whole litters. As it was expected that piglets within each litter would influence each other's behaviours, the litter was used as the unit of measurement for the behavioural comparisons and not the individual piglet, thus behavioural values were the average of those piglets observed within each litter. As with previous studies in this housing system [29] group size was not found to associate with expression of behaviours of interest. Behavioural data was normally distributed and not transformed for analysis.

## 3 Results

### 3.1 Comparisons between home pen and arena behaviour

**Fig. 2:**
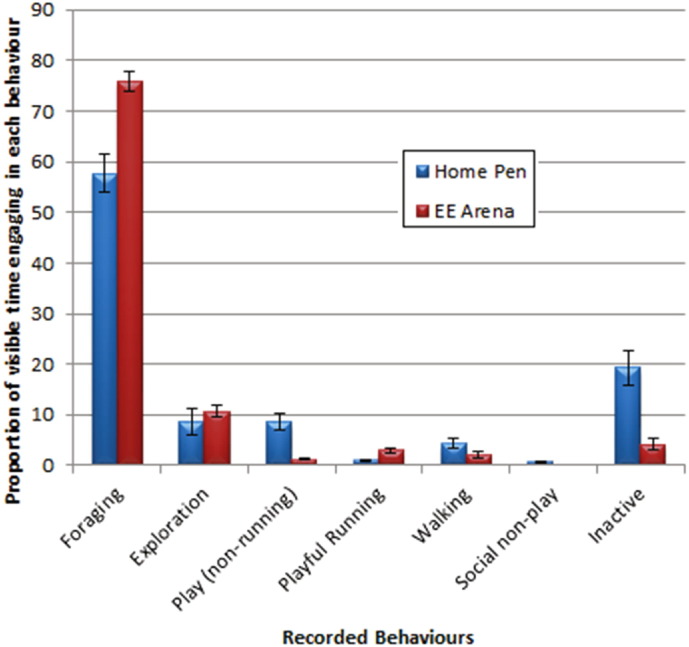
Proportion of visible time (time when animal was in sight of recording equipment during the observational phase) spent engaging in foraging, exploration, playful running, non-running play, walking and inactivity in both the home pen (blue) and the EE arena (red). Home pen observations were taken over a 2 h period during week 2 post weaning with enriched arena observations taken over a 15 min period at the same time of day during week 4 post weaning; the longer period of home pen observation was to allow for sampling of spontaneous behaviours. Only piglets observed in the arena were observed in the home pen. Error bars show standard errors of the mean.

Overall piglets spent the largest proportion of visible time in the home pen performing foraging and consummatory behaviour (home pen mean = 57.64%, SEM = 3.53) with a significant increase (*t*_7_ = 5.35, *p* = 0.001) in these behaviours in the EE arena (EE arena mean = 75.9%, SEM = 1.9). Playful running was also found to increase in the EE arena (home pen mean = 0.98%, SEM = 0.25; EE arena mean = 2.94%, SEM = 0.496; *t*_7_ = 3.62, *p* = 0.009) though this does not reach significance with the multiple testing correction applied. Non-running play (home pen mean = 8.51%, SEM = 1.62; EE arena mean = 1.21%, SEM = 0.16; *t*_7_ = 4.82, *p* = 0.002) and percentage of time spent inactive (home pen mean = 19.38%, SEM = 3.46; EE arena mean = 4.21%, SEM = 1.25; *t*_7_ = 4.60, *p* = 0.002) were found to decrease in the EE arena. Exploratory behaviour (home pen mean = 8.5%, SEM = 2.61; EE arena mean = 10.75%, SEM = 1.12) and walking (home pen mean = 4.31%, SEM = 0.97; EE arena mean = 2.09%, SEM = 0.57) were not found to differ between home pen and EE arena. A small amount (mean = 0.68%, SEM = 0.23) of social non-play behaviours occurred in the home pen but not in the EE arena. These included potentially aggressive encounters (Fig. 2).

### 3.2 Frontal cortex gene expression

**Fig. 3:**
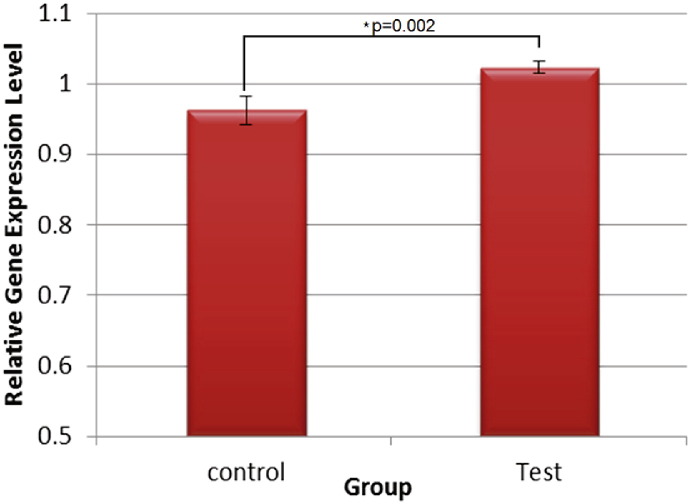
Relative expression of *IGF-1* mRNA in frontal cortex of piglets within 45 min of a 15 min exposure to the EE arena (test) and within 24 h of a 15 min exposure to the EE arena (control).

A significant fold change (FC) increase (FC = 1.07, *t*_7_ = 4.42, *p* = 0.002) was observed in *IGF-1* gene expression in the frontal cortex of piglets exposed to EE compared to those not exposed on the day of culling (Fig. 3).

No change in expression was observed in the *IGF-1* receptor gene nor in any of the genes for the binding proteins tested (*IGFBP1-6*) (Table 3). There was a weak tendency for increased expression of the neurotrophic factor *BDNF1* (FC: 1.03; *t*_7_ = 1.54, *p* = 0.1).

## 4 Discussion

The main aim of this paper was to analyse the effects of exposure to short-term environmental enrichment (EE) on species typical behaviour and brain gene expression in weaned piglets. As the pig is physiologically closer to the human than standard rodent models [22], [30], this study gives insight into the effects of EE exposure on gene expression levels in the frontal cortex of larger mammals for which there is little, if any, information. The use of EE has for a long time been proposed as an intervention to improve standards of livestock care (*e.g.* [1]) however little has been done to determine the efficacy of a short term exposure to EE in pigs in terms of behaviour and brain effects.

In mice, genes involved in neuronal structure, synaptic signalling, and plasticity have been shown to be altered in the cortex after 3 and 6 h of environmental enrichment suggesting early molecular events arising from EE include strengthening and modifying of cortical synapses [31]. Among these neural implications of EE, the expression of growth factors in brain tissues has been repeatedly shown to be altered in rodent models of EE (reviewed in Van Praag et al. 2000 [2]), most prominently through the upregulation of Insulin like growth factor (*IGF-1*) and Brain Derived Neurotrophic factor (*BDNF*). Rats with experience of EE show higher numbers of IGF-1 positive neurones in the developing visual cortex than those with no EE [32], and IGF-1 signalling has been proposed as one of the underlying mechanisms by which EE imposes beneficial effects on brain recovery following cerebral ischaemic injury in a rat model [33].

IGF-1 has previously been described as a protein linking ‘body and brain fitness’ as it is significantly involved in physical growth while also having important brain effects including previously mentioned involvement in neuronal ‘health’, and in positive emotional states [34]. *IGF-1* expression in the brain has been previously shown to be upregulated in the frontal cortex of rats after a play experience but not after social defeat [19], indicating a possible role in modulating affect. Indeed in rats, injection of IGF-1 early in life mimicked the effects of early life experience of EE when animals were run through an anxiety paradigm. The reverse was also true in that blocking IGF-1 action early in life in animals housed under EE conditions negated the effects of the EE condition on later anxiety levels [35]. In humans circulating levels of IGF-1 peptide correlate negatively with symptoms of low mood, depression and anxiety [36], [37] so expression levels of IGF-1 may be a useful indicator of positive affect.

IGF-1 has 6 known binding partners, of which all exhibit an inhibitory effect on IGF-1 action, with 3 binding proteins (1, 3 and 5) also exhibiting a stimulatory effect. These 3 ‘multipurpose’ binding partners also exhibit IGF-1 independent effects including inhibition of cell growth and induction of apoptosis (reviewed in [38]). A recent study of human Alzheimers patients has identified an increase in astrocyte production of IGF binding protein 3 (IGFBP3) as a contributory factor to Alzheimers disease pathology [39]. Interestingly, *IGFBP3*, which had previously been shown to have altered expression in the cortex post play experience in rats [19], did not show any differences in this study population of pigs after EE. There are a number of possible reasons this could be the case. Firstly the piglets in this study were culled within 45 min of enrichment, while the rats in [19] were culled 6 h post play experience, and there could be a temporal delay in *IGFBP3* expression relative to *IGF-1*. Secondly, to elicit the species typical rough and tumble play in rats they are socially isolated whereas the piglets were moved in pairs between the home pen and the enriched arena. Humans who experience subjective social isolation are known to have higher expression of *IGFBP3* from peripheral sampling (leukocytes) [40] and while it is difficult to compare across tissue samples it could be that the elevated *IGFBP3* reported by [19] may be an effect of social isolation, as in the above human study, and not a result of up-regulation after play behaviour. This current study attempted to minimise the effect of isolation from littermates on gene expression and behaviour, though it could be argued that the change in group dynamics would affect the piglet even in a short duration trial. However, as the piglets not tested on the day of tissue collection also, ultimately, experienced a change in group dynamics through the removal of 4 littermates this effect is likely to be negligible when comparing between the 2 ‘treatments’.

Conversely brain derived neurotrophic factor (*BDNF*), which was not identified as showing altered expression in [19], showed a trend towards up-regulation in the piglet post EE exposure. The interaction between *IGF-1* and *BDNF* in exercise induced gene expression change and brain plasticity is well known [34] and other recent studies have shown that in rats an elevated *BDNF* response in the hippocampus to an inescapable foot shock is also enhanced in animals housed in enriched environments post-weaning [41] suggesting enriched housing may induce a neuroprotective state in the longer term [42] through the actions of BDNF. Thus enriched housing may provide a solution for long term physical and mental well-being. As the current study focussed on short term exposure and did not look at longer term effects it is possible a significant effect of EE on *BDNF* expression may have been missed. Contrary to our original hypothesis, the EE paradigm in this study had no effect on expression of the *CSF1* gene in the frontal cortex suggesting the increase in *IGF-1* may not be due to local microglial expression in response to *CSF1* [43]. However, it is important to note the current study compares samples from pigs which have all had EE exposure, with the controls having access to the arena 24 h prior to sample collection and the test animals 45 min prior to sample collection. So any changes in gene expression in this study population can, in the absence of a naïve control, only be interpreted as a short term upregulation immediately post EE, as we can neither confirm nor deny whether the controls differ from individuals whom had never experienced the EE arena. Nevertheless this is the first evidence of an up-regulation of *IGF1* (and *BDNF*) gene expression in the brain of a large mammal immediately following EE.

There is very little information in the literature on the effects of EE on spontaneous behaviour in rodents. The limited studies available show that rats raised in enriched environments show reduced exploration and rapid locomotory habituation [44] using a Behaviour Pattern Monitor (BPM) paradigm (as described in [45]) suggesting a rapid assimilation of information about their environment, while socially isolated animals display an increase in exploration and a reduction in PrePulse Inhibition suggestive of an impairment in sensory motor gating. Pigs have evolved as ground foragers and free-ranging domestic pigs will spend up to 55% of their daylight hours foraging [46] while commercially housed indoor pigs are seen to perform foraging behaviour significantly less often [47], [48]. It is therefore interesting that in this study the home pen foraging behaviours averaged those of free-ranging pigs in previous studies with behaviour increasing further in the enriched arena. Piglets were housed in a high welfare system and already allocated a rooting substrate (straw) in the home pen with their nutritional needs being met with *ad libitum* provision of a standard diet, therefore the act of rooting/foraging provided no nutritional reward to the piglets in this system, rather the action itself must have been self-rewarding. Previous work on the motivation of chewing and exploration behaviours in growing pigs has also found that chewing of a novel object occurs independently of an underlying feeding motivation [49]. The observation of previous studies that rooting/foraging is reduced in sows after administration of naloxone [50] would support the suggestion that foraging behaviour is intrinsically rewarding to the pig. It could then be that the allocation of an alternative substrate (peat and straw mix) in the enriched arena may have further increased the pigs' intrinsic motivation to root and forage when given the opportunity [51] even when their behavioural and physiological needs were adequately met in their home pen. Previous studies have shown that pigs will perform more rooting behaviour in a newly allocated area even when given constant access to outdoor grazing areas [52] so this changing of substrate may be a valuable tool in providing enrichment for growing pigs. This has potential implications not only in livestock management but in human health, where reward responsiveness at the molecular level may be affected by EE [53], [54].

The increase in locomotor play behaviours such as running when in the enriched arena is perhaps not surprising given the increased space allowance (per piglet) [55], [56]. Similarly the decrease observed in inactivity would be expected given the provision of a more stimulating environment [56]. What was perhaps surprising was the lack of change in other non-running play behaviours such as pivots and hops which have previously been associated with a positive welfare state in pigs [55] and have been proposed as potential indicators of positive welfare [55], [29]. However previous studies did not look at these behaviours in the presence of new enrichment substrates, therefore it is difficult to ascertain if in this study population the motivation to forage was greater than the motivation to perform non-running play behaviours in the new environment. Given the time in the arena was short, and novel resources were available (when compared to the home pen substrate), it could be postulated the piglets prioritised the foraging behaviour above others, especially as running and walking behaviours combined made up on average only 5% of the time budget in the arena. The lack of association between behaviours in the home pen and the enriched arena would suggest that the spontaneous behaviours of the home pen and the induced behaviours of the arena are not predictive of one another. Future work to determine if there is an imposable ‘upper limit’ to substrate enrichment, and assessment to determine preferred substrates would be beneficial [57].

## 5 Conclusions

This study is the first to show molecular changes in the brain of large mammals exposed to EE in addition to an increase in behaviours (rooting/foraging) which have the characteristics of being self-rewarding. When viewing the observation of increased *IGF-1*, and a tendency for increased *BDNF*, in piglets after a brief exposure to EE alongside previous rodent and human studies of increased *IGF-1* correlating with learning, mood and recovery from brain injury, it would suggest that in pigs short-term exposure to EE results in: 1) increased positive affect; 2) increases in synaptogenesis and plasticity, with concomitant beneficial effects on learning, memory and cognitive development and 3) increases in neuroprotection with prolonged positive effects on ‘brain health’.

## Figures and Tables

**Table 1:** Ethogram used in both the home pen and enriched arena for behavioural scoring.

Behaviour	Definition/type	References
Foraging	Any event where nose of piglet makes contact with the straw or peat including rooting and grazing.	Defined for this study
Exploration	Nose making contact with either the bare floor of the apparatus or walls (often combined with walking).	Defined for this study
Playful running	Energetic running and hopping in forward motions often associated with excitability, using large areas of the pen, and occasionally coming into marginal/accidental contact with other piglets (*e.g.* nudge).	[29]
Play (non-running)	Pivots, flops, hops, gentle nudging and moderate pushing of a pen mate.	[29]
Social non-play	Pushing, biting, head knocking and potentially harmful fights.	Defined for this study
Walking	A steady 4 beat gait with 2 or 3 legs bearing weight at any one time (depending on the phase of the movement).	Defined for this study
Inactive	Ventral or lateral lying with no movement.	Defined for this study
Escape	Placing one or more feet on the top of any side of the apparatus and attempting to escape.	Defined for this study

**Table 2:** Taqman probes used for quantitative PCR analysis. Left hand column gives gene target name with the Applied Biosystems catalogue number in the right hand column. Middle column gives the sequence interrogated for probe development by ABI (Refseq or GenBank ID). Probes were of standard inventoried stock for amplification of pig genes.

Probe ID (gene target name)	Interrogated sequence	Catalogue number
(Actb)	AK237086.1	Ss03376081_u1
(Hprt1)	NM_001032376.2	Ss03388274_m1
(IGF1)	NM_214256.1	Ss03394499_m1
(IGF1R)	NM_214172.1	Ss03394286_m1
(IGFBP1)	NM_001195105.1	Ss03374977_u1
(IGFBP2)	NM_214003.1	Ss03393382_u1
(IGFBP3)	J05228.1	Ss03374257_u1
(IGFBP4)	NM_001123129.1	Ss03387801_u1
(IGFBP5)	NM_214099.1	Ss03382569_u1
(IGFBP6)	NM_001093660.1	Ss03386322_u1
(BDNF)	NM_214259.2	Ss03822335_s1
(CSF1)	AJ583705.1	Ss03373560_g1

**Table 3:** Relative gene expression values for genes tested in piglet frontal cortex. Columns show approved gene symbols, fold change from control to test animals, *t* statistic and *p* value of the paired *t*-test.

Gene of interest	Fold change	*t*-test	*p* value	N (per group)
IGF-1	1.07	4.42	0.002	8
BDNF	1.03	1.540	0.10	8
IGF1R	− 1.11	0.824	0.424	8
IGFBP1	1.01	0.358	0.726	8
IGFBP2	1.01	0.2491	0.8069	8
IGFBP3	− 1.01	0.444	0.6637	8
IGFBP4	− 1.03	0.073	0.9429	8
IGFBP5	1.02	0.386	0.7054	8
IGFBP6	1.01	0.322	0.752	8
CSF1	1.00	0.205	0.84	8
